# Low serum vitamin D concentration is correlated with anemia, microinflammation, and oxidative stress in patients with peritoneal dialysis

**DOI:** 10.1186/s12967-021-03077-w

**Published:** 2021-09-27

**Authors:** Chi Zhang, Junsheng Wang, Xiaohui Xie, Dong Sun

**Affiliations:** 1 Department of Nephrology, The Affiliated Suqian Hospital of Xuzhou Medical University, No. 138 Huanghe Road South, Suqian, 223800 Jiangsu China; 2 Xuzhou Medical University, No. 99 West Huai-hai Road, Xuzhou, 221002 Jiangsu China; 3 Department of Internal Medicine and Diagnostics, Xuzhou Medical University, No. 99 West Huai-hai Road, Xuzhou, 221002 Jiangsu China

**Keywords:** Peritoneal dialysis, Vitamin D, Anemia of chronic kidney disease, Oxidative stress, Microinflammation

## Abstract

**Background:**

Peritoneal dialysis (PD) is a form of dialysis to replace the function of kidney, that uses the peritoneum as a dialysis membrane to remove metabolites and water retained in the body. Vitamin D deficiency is prevalent in patients treated with PD. This research investigated the correlation between serum 25-hydroxyvitamin D [25(OH)D] concentration and anemia, microinflammation, and oxidative stress in PD patients.

**Methods:**

62 PD patients and 56 healthy volunteers were recruited in this research. Serum concentrations of 25(OH)D and basic parameters of anemia were detected. The correlation between serum 25(OH)D concentration with anemia, oxidative stress, and microinflammatory state were analyzed.

**Results:**

In the PD group, the concentration of 25(OH)D was lower than the healthy control (HC) group (p < 0.001). Hemoglobin, red blood cell count (RBC), and total iron binding capacity (TIBC) in the PD group was significantly lower (all p < 0.001), while high-sensitivity C-reactive protein (hs-CRP), interleukin-6 (IL-6), and tumor necrosis factor α (TNF-α) concentrations were significantly higher, than the HC group (all p < 0.001). In the PD group, malondialdehyde (MDA) concentration was higher than in the HC group (p < 0.001), while superoxide dismutase (SOD) and glutathione peroxidase (GSH-Px) were lower (both p < 0.001). Serum 25(OH)D exhibited positive correlation with hemoglobin (r = 0.4509, p = 0.0002), RBC (r = 0.3712, p = 0.0030), TIBC (r = 0.4700, p = 0.0001), SOD (r = 0.4992, p < 0.0001) and GSH-Px (r = 0.4312, p = 0.0005), and negative correlation with hs-CRP (r = − 0.4040, p = 0.0011), TNF-α (r = − 0.4721, p = 0.0001), IL-6 (r = − 0.5378, p < 0.0001) and MDA (r = − 0.3056, p = 0.0157).

**Conclusion:**

In conclusion, reduced serum 25(OH)D concentrations in PD patients contribute to anemia, oxidative stress and microinflammatory state.

**Supplementary Information:**

The online version contains supplementary material available at 10.1186/s12967-021-03077-w.

## Background

Peritoneal dialysis (PD) is a form of dialysis to replace the function of kidney, that uses the peritoneum as a dialysis membrane to remove metabolites and water retained in the body [1]. Recent studies have found that as the duration of PD increases, the homeostasis of the body’s internal environment is disrupted, and systemic inflammatory responses and oxidative stress gradually appear, forcing some patients to discontinue PD due to serious complications [2–4].

In patients, microinflammation is a state of sustained low-level inflammation, clinically manifested by the elevated levels of inflammatory factors [5]. Microinflammation is mostly mediated by intravascular inflammation caused by relative inflammatory substances, and its impact on the patient is manifested in several ways, closely related to anemia, malnutrition and low quality of life [6]. A high prevalence of various cardiovascular events is characteristic of end-stage renal diseases and is common in dialysis patients [7]. Existing studies have shown that the early intervention of microinflammatory status can inhibit cardiovascular complications and alleviate anemia and malnutrition status in hemodialysis patients [8]. However, studies on the effect of early intervention of microinflammation on PD patients are still limited.

Patients with lower serum 25-hydroxyvitamin D [25(OH)D] concentrations were treated with PD. Reasons for vitamin D deficiency in PD patients include chronic renal dysfunction, reduced sunlight exposure, restricted dietary and peritoneal effluent [9, 10]. Vitamin D deficiency is reported to correlate with enhanced inflammation in stable hemodialysis patients [11].

This research aimed to explore the relationship between vitamin D deficiency and anemia, microinflammation and oxidative stress in PD patients.

## Methods

### Patients

In this research, 62 patients who received PD in the Affiliated Suqian Hospital of Xuzhou Medical University were selected as the PD group, and 56 healthy volunteers were recruited as the healthy control (HC) group. All patients provided written informed consent. Inclusion and exclusion criteria were shown in Additional file 1: Figure S1. This research was approved by the Ethics Committee of the Affiliated Suqian Hospital of Xuzhou Medical University.

### Measurements

Blood samples were collected and stored at – 80 °C. The concentration of 25(OH)D was analyzed using Roche Cobas E601 ECL analyzer (Roche, Geneva, Switzerland) and Roche Cobas Vitamin D total assay reagent (Roche Diagnostics GmbH, Mannheim, Germany). Calibration curves were constructed using calibrators provided in the kits.

Hemoglobin and red blood cell count (RBC) were measured through the UniCel DxH 600 Coulter Cellular Analysis System hematology analyzer (Beckman Coulter, Miami, FL). Total iron binding capacity (TIBC) was measured by AU5810 Chemistry Analyzers (Beckman Coulter).

Serum concentrations of high-sensitivity C-reactive protein (hs-CRP), interleukin-6 (IL-6) and tumor necrosis factor α (TNF-α) were detected with high-sensitivity enzyme-linked immunosorbent assay kits (R&D Systems, Minneapolis, USA). Malondialdehyde (MDA) concentration was determined through thiobarbituric acid method and superoxide dismutase (SOD) concentration was determined by pyrogallol autoxidation method, and the kits were purchased from Sichuan Vichy Biotechnology Co. Serum glutathione peroxidase (GSH-Px) concentration was determined by fluorometric assay, and the kit was purchased from Shanghai Yuanye Biotechnology Co. All the internal controls were provided by the kits.

### Statistical analysis

SPSS 22.0 was used for data analysis. Data were expressed as median with interquartile range or n (percentage, %). Differences for the two groups were compared using Mann–Whitney test. Proportions were compared using Chi-square (χ^2^) test. Linear correlations were verified using the Spearman’s correlation analysis. Receiver operating characteristic (ROC) analyses was employed to analyze the predictive value of serum 25(OH)D level on the state of peritoneal dialysis patients. The probability p < 0.05 was considered as the minimum condition of statistical significance.

## Results

### Demographics and clinical characteristics

The demographics and clinical characteristics of the participants were shown in Table 1. Based on the results of statistical analyses, these two groups were homogenous for age, gender, body mass index, blood pressure, proportion of diabetes mellitus and cardiovascular disease, and the concentrations of serum albumin, triglycerides, and cholesterol (all p > 0 0.05). The median dialysis duration was 27 (19–39) months (Table 1). Chronic glomerulonephritis was the most frequent primary kidney disease (48.4%), followed by diabetic nephropathy (24.19%) and hypertensive nephropathy (9.7%) (Table 1).

**Table 1:** Demographics and clinical characteristics of the peritoneal dialysis patients and healthy controls

	Study group		p
	PD (n = 62)	HC (n = 56)	
Age (years)	54 (46–64)	52 (45–61)	0.52
Gender (%)			
Male	35 (56.5%)	29 (51.8%)	0.71
Female	27 (43.5%)	27 (48.2%)	
BMI (kg/m^2^)	22 (20–27)	23 (20–28)	0.17
Systolic blood pressure (mmHg)	142 (132–156)	126 (109–134)	0.03
Diastolic blood pressure (mmHg)	86 (75–102)	75 (69–86)	0.02
Dialysis duration (months)	27 (19–39)	–	–
Diabetes mellitus (%)	17 (27.4%)	11 (19.6%)	0.39
Cardiovascular disease (%)	14 (22.6%)	7 (12.5%)	0.23
Primary kidney disease (%)			
Diabetic nephropathy	15 (24.2%)	–	–
Hypertensive nephropathy	6 (9.7%)	–	
Chronic glomerulonephritis	30 (48.4%)	–	
Others	11 (17.7%)	–	
Serum albumin (g/L)	31 (26–38)	44 (39–51)	0.004
Triglycerides (mmol/L)	2.4 (1.9–3.2)	1.3 (0.9–2.1)	0.02
Cholesterol (mmol/L)	4.8 (3.6–5.4)	3.9 (3.2–4.7)	0.09
MCV (fL)	80.6 (73.2–84.9)	89.4 (82.8–97.3)	0.005
Hematocrit (%)	37.6 (33.7–41.3)	43.8 (40.5–46.1)	0.01

Values were expressed as Median with interquartile range or n (%)

p values for each group were derived from Chi-square test or Mann–Whitney test

*PD* peritoneal dialysis, *HC* healthy controls, *BMI* body mass index, *MCV* mean corpuscular volume

### 25(OH)D concentrations are downregulated in PD patients

**Fig. 1:**
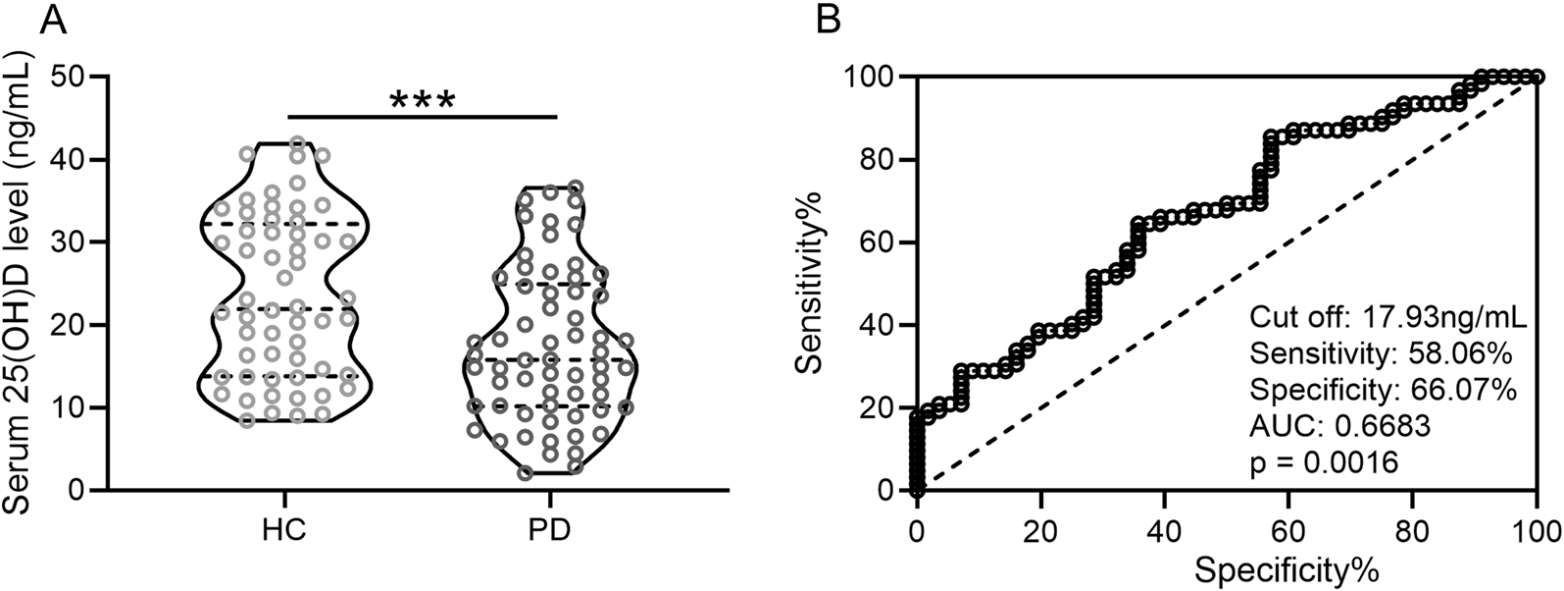
Serum 25(OH)D concentrations were downregulated in patients with PD. **A** Serum 25(OH)D concentrations in both groups were analyzed. **B** ROC analysis of serum 25(OH)D concentrations. Violin plot was used to show the data. ***p < 0.001, Mann–Whitney test

In this research, we first analyzed the serum concentration of 25(OH)D in both groups. As shown in Fig. 1A, the median concentration of 25(OH)D was 15.8 (10.2–24.9) ng/mL in the PD group and 21.9 (13.8–32.2) ng/mL in the HC group. Thus, the PD group showed significantly lower 25(OH)D concentration than the HC group (p = 0.0009) (Fig. 1A). Figure 1B showed the ROC curve analysis for serum 25(OH)D. The area under the curve was 0.6683 (p = 0.0016) and cut-off concentration was 17.93 ng/mL, with 66.07% specificity and 58.06% sensitivity (Fig. 1B).

### Low serum 25(OH)D concentration is correlated with anemia in patients with PD

**Fig. 2:**
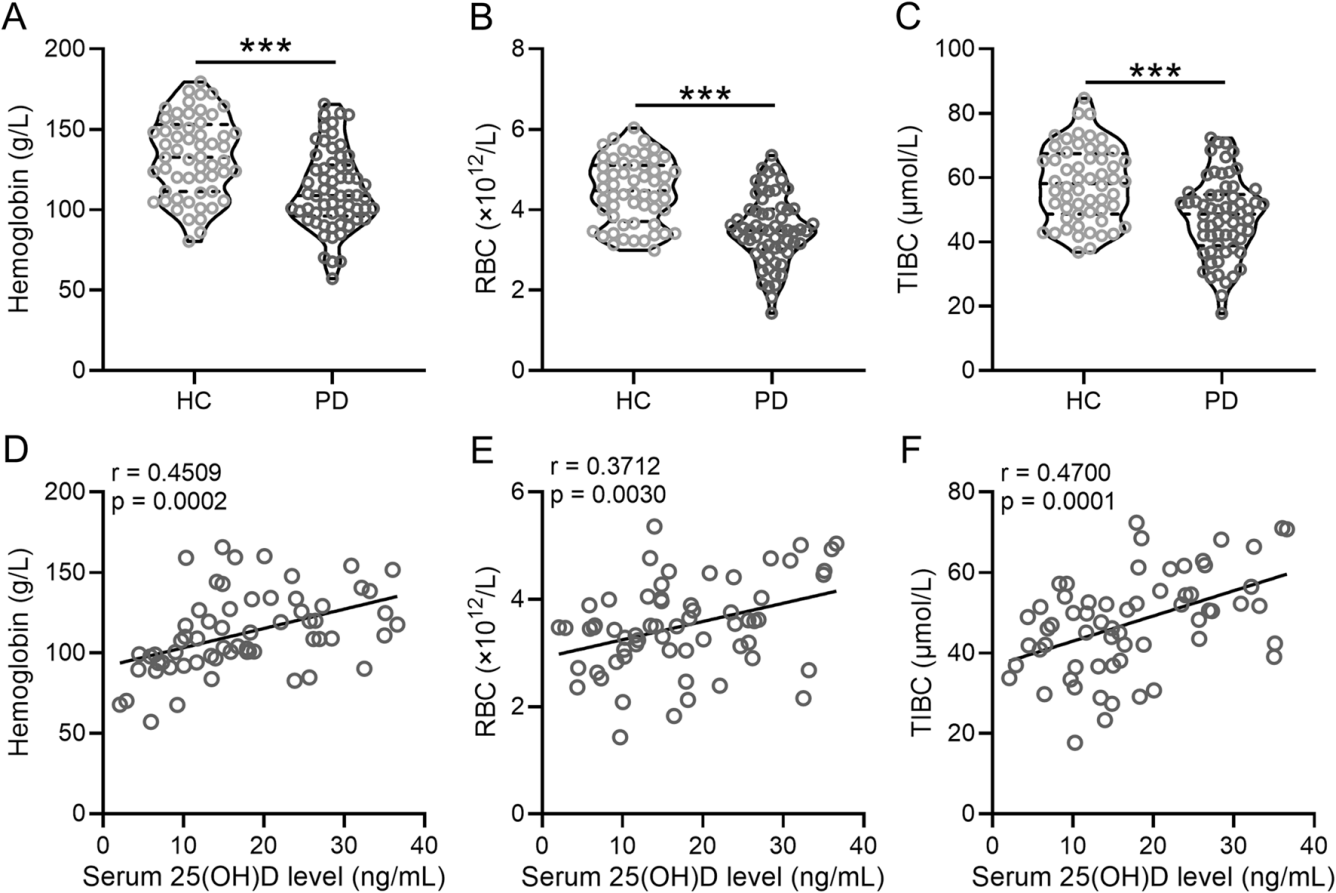
Low serum concentrations of 25(OH)D are correlated with anemia in patients with PD. Concentrations of hemoglobin (**A**), RBC (**B**), and TIBC (**C**) were compared between PD and healthy controls. Violin plot was used to show the data. Spearman correlation analysis of serum concentrations of 25(OH)D with hemoglobin (**D**), RBC (**E**), and TIBC (**F**). ***p < 0.001, Mann–Whitney test

Comparison between patients from the two groups revealed that the PD group displayed significantly lower hemoglobin (109 (96–128) g/L VS 133 (111–153) g/L; p < 0.0001), RBC (3.5 (3–4) × 10^12^/L VS 4.5 (3.7–5.1) × 10^12^/L; p < 0.0001), and TIBC levels (49 (39–55) μmol/L VS 58 (49–67) μmol/L; p < 0.0001) than the HC group (Fig. 2A–C, Additional file 1: Table S1). Furthermore, we also analyzed whether vitamin D deficiency was correlated with decreased hemoglobin, RBC and TIBC in PD patients. Spearman correlation analysis indicated that 25(OH)D concentration exhibited positive correlation with hemoglobin (r = 0.4509; p = 0.0002), RBC (r = 0.3712; p = 0.0030) and TIBC (r = 0.4700; p = 0.0001) in patients with PD (Fig. 2D-F).

### Low serum 25(OH)D concentration is correlated with microinflammation in patients with PD

**Fig. 3:**
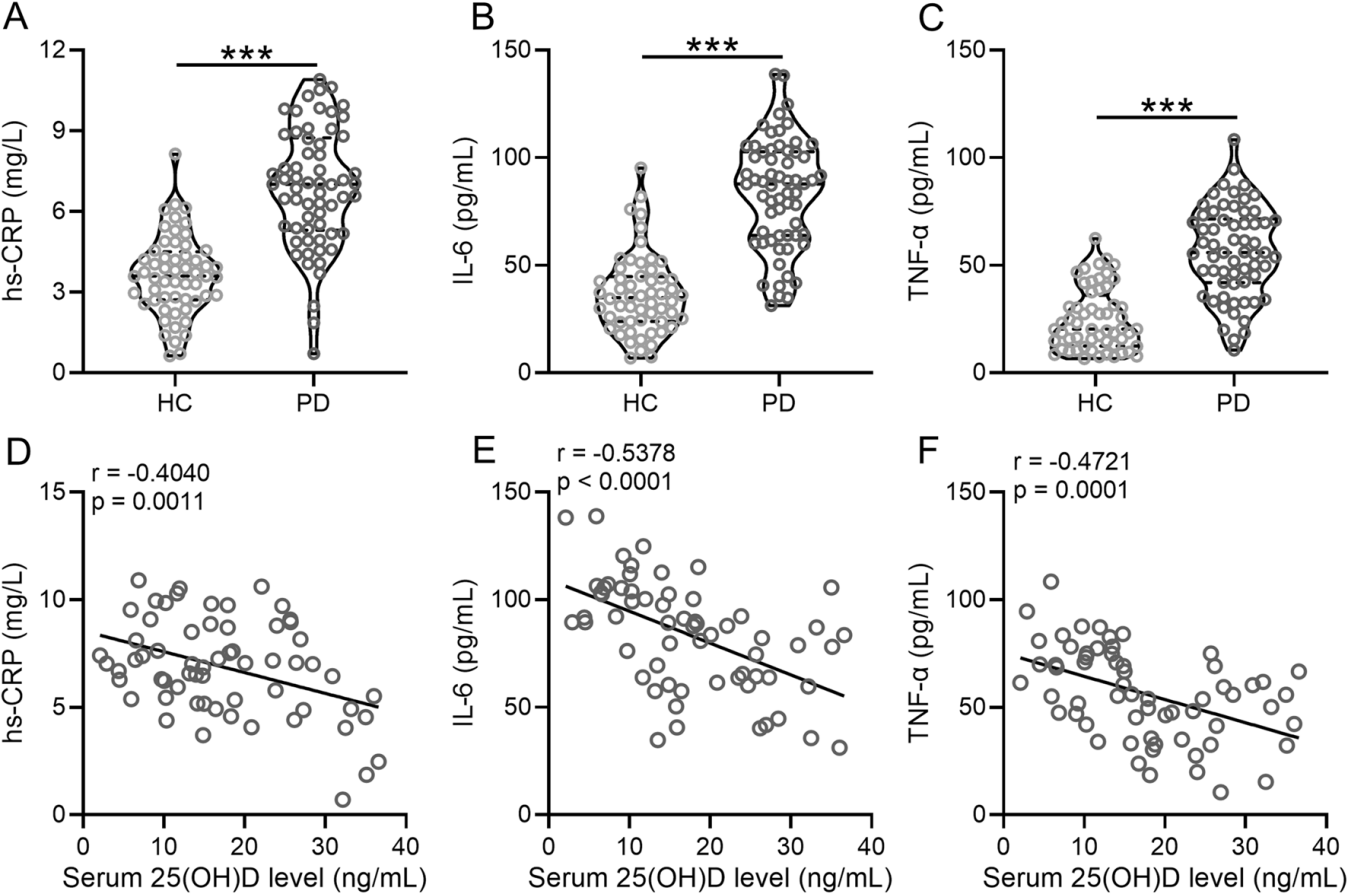
Low serum concentrations of 25(OH)D are correlated with microinflammation in patients with PD. Concentrations of hs-CRP (**A**), IL-6 (**B**), and TNF-α (**C**) were compared between PD and healthy controls. Violin plot was used to show the data. Spearman correlation analysis of serum concentrations of 25(OH)D with hs-CRP (**D**), IL-6 (**E**), and TNF-α (**F**). ***p < 0.001, Mann–Whitney test

Comparison between patients from the two groups revealed that the PD group exhibited significantly higher concentrations of hs-CRP [7 (5.3–8.7) mg/L VS 3.6 (2.7–4.5) g/L; p < 0.0001], IL-6 [88 (64–103) pg/mL VS 35 (24–45) pg/mL; p < 0.0001], and TNF-α [56 (42–71) pg/mL VS 20 (12–36) pg/mL; p = 0.0047] than the HC group (Fig. 3A–C, Additional file 1: Table S1). Spearman correlation analysis showed that 25(OH)D concentration was negatively correlated with hs-CRP (r = − 0.4040; p = 0.0011), IL-6 (r = − 0.5378; p < 0.0001) and TNF-α (r = − 0.4721; p = 0.0001) concentrations in patients with PD (Fig. 3D–F).

### Low serum 25(OH)D concentration is correlated with oxidative stress in patients with PD

**Fig. 4:**
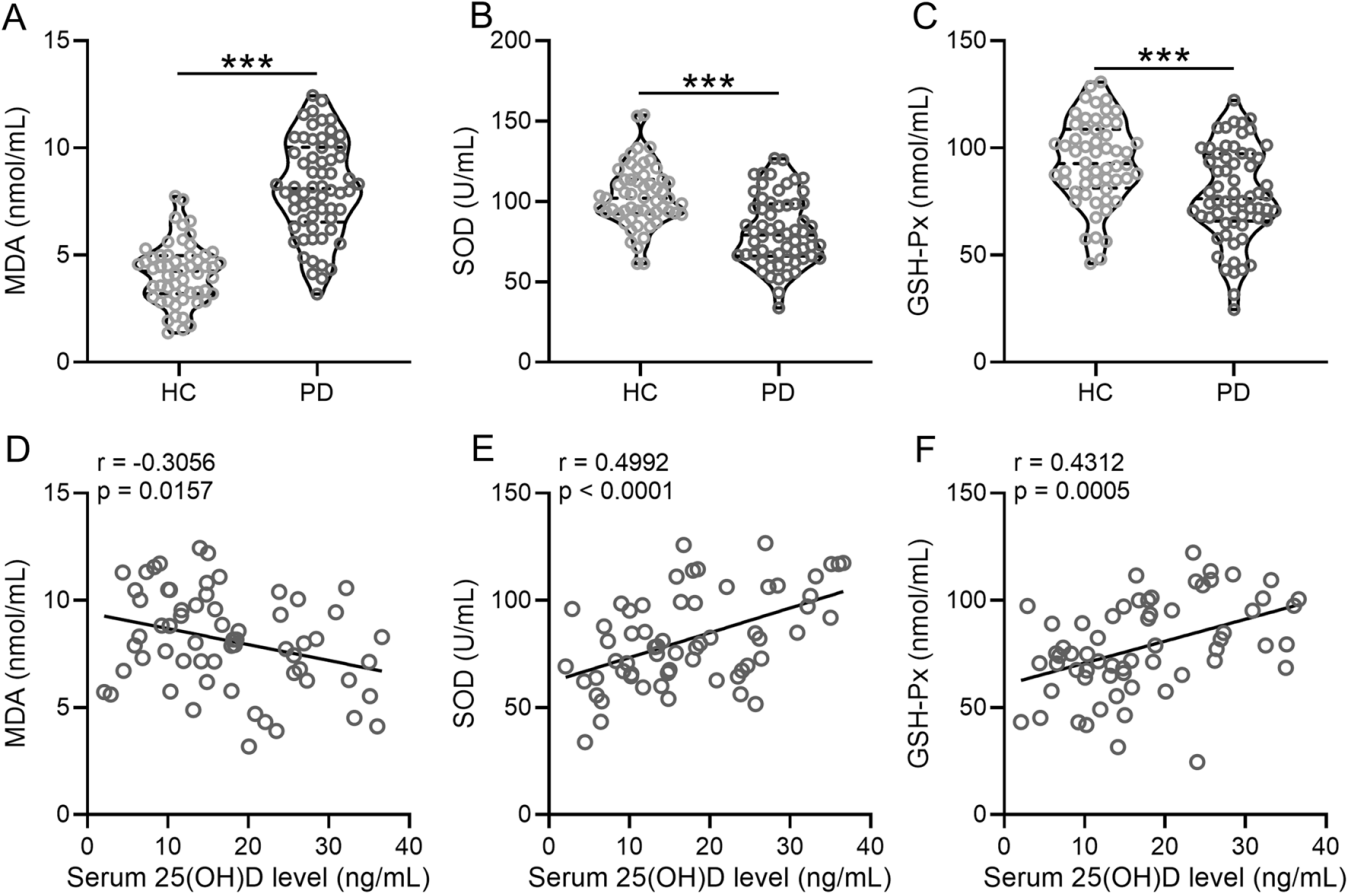
Low serum concentrations of 25(OH)D are correlated with oxidative stress in patients with PD. Concentrations of MDA (**A**), SOD (**B**), and GSH-Px (**C**) were compared between PD and healthy controls. Violin plot was used to show the data. Spearman correlation analysis of serum concentrations of 25(OH)D with MDA (**D**), SOD (**E**), and GSH-Px (**F**). ***p < 0.001, Mann–Whitney test

Comparison between patients from the two groups revealed that the HC group showed significantly lower concentration of MDA [4.2 (3.2–4.9) nmol/mL VS 8.1 (6.5–10) nmol/mL; p < 0.0001], and higher in the levels of SOD [102 (92–114) U/mL VS 79 (66–99) U/mL; p < 0.0001], and GSH-Px (93 (81–109) nmol/mL VS 76 (66–97) nmol/mL; p = 0.0002), than the PD group (Fig. 4A–C, Additional file 1: Table S1). Spearman correlation analysis indicated that serum 25(OH)D concentration was negatively correlated with MDA (r = − 0.3056; p = 0.0157), while positively correlated with SOD (r = 0.4992; p < 0.0001) and GSH-Px (r = 0.4312; p = 0.0005) concentrations in patients with PD (Fig. 4D–F).

### Low serum 25(OH)D concentration is correlated with anemia, microinflammation and oxidative stress in PD patients

**Fig. 5:**
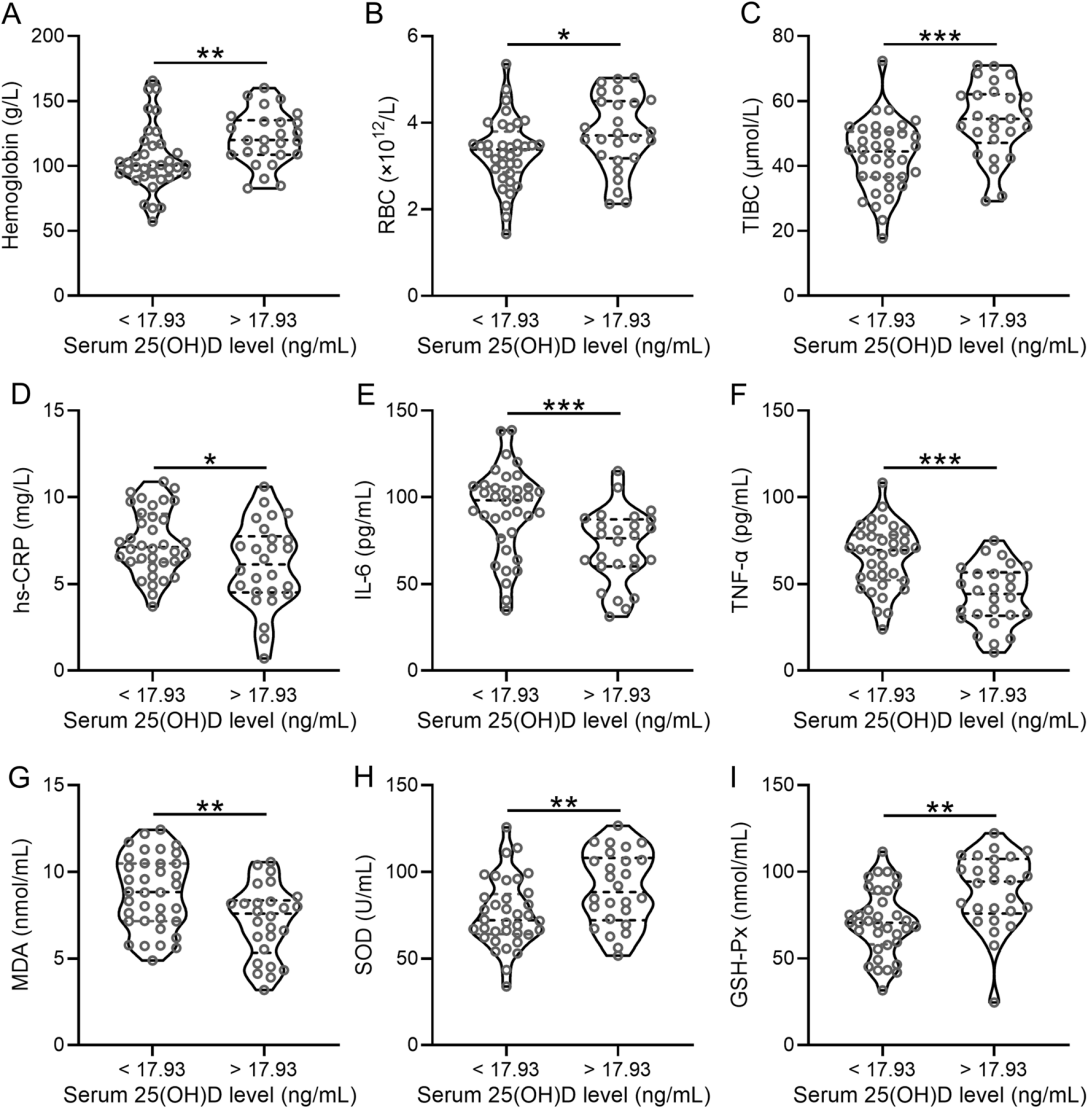
Low serum concentrations of 25(OH)D are correlated with anemia, microinflammation and oxidative stress in patients with PD. The cut off was set by ROC analysis and concentrations of hemoglobin (**A**), RBC (**B**), TIBC (**C**), hs-CRP (**D**), IL-6 (**E**), TNF-α (**F**), MDA (**G**), SOD (**H**), and GSH-Px (**I**) were compared between high 25(OH)D concentration (> 17.93 ng/mL, n = 26) and low 25(OH)D concentration (< 17.93 ng/mL, n = 36). Violin plot was used to show the data. *p < 0.05, **p < 0.01, ***p < 0.001, Mann–Whitney test

Based on the cut-off value of serum 25(OH)D concentration, patients with PD were divided into high concentration group (> 17.93 ng/mL) and low concentration group (< 17.93 ng/mL). Patients with high 25(OH)D concentration showed significantly higher hemoglobin [120 (109–135) g/L VS 100 (92–117) g/L; p = 0.0080], RBC [3.7 (3.2–4.5) × 10^12^/L VS 3.4 (2.9–3.8) × 10^12^/L; p = 0.0468], TIBC [54 (47–62) μmol/L VS 44 (37–51) μmol/L; p = 0.0003], SOD [88 (72–108) U/mL VS 72 (64–87) U/mL; p = 0.0086], and GSH-Px [94 (76–108) nmol/mL VS 71 (58–87) nmol/mL; p = 0.0015] (Fig. 5A–C, H, I; Additional file 1: Table S1). On the other hand, patients with high 25(OH)D concentration showed significantly lower concentrations of hs-CRP [6.1 (4.5–7.7) g/L VS 7.1 (6.2–9.1) mg/L; p = 0.0256], IL-6 [76 (60–87) pg/mL VS 98 (77–106) pg/mL; p = 0.0009], TNF-α [44 (32–57) pg/mL VS 69 (52–78) pg/mL; p < 0.0001], and MDA [7.6 (5.3–8.4) nmol/mL VS 8.8 (7.2–10.5) nmol/mL; p = 0.0028] than those with low 25(OH)D concentration (Fig. 5D–G, Additional file 1: Table S1).

## Discussion

Consistent with previous studies, we found that, compared with the healthy control group, PD patients showed lower 25(OH)D concentrations in the peripheral blood. Vitamin D deficiency can be observed in most dialysis patients and is usually associated with malnutrition [12, 13]. Vitamin D has been shown to play a role in the regulation of the innate and adaptive immune systems [14, 15]. Vitamin D deficiency has been shown to be associated with an increased risk of PD-associated peritonitis [12]. Intervention studies have also shown that oral vitamin D supplementation can reduce the rate of respiratory infections [16]. There is evidence supporting that 25(OH)D deficiency is also associated with an increased risk of anemia [17]. In adults with chronic kidney diseases, lower 25(OH)D concentrations are associated with lower hemoglobin concentrations and anemia, and vitamin D has also been shown to play a role in erythropoiesis [18]. It has been reported that PD patients with low serum 25(OH)D concentrations have a reduced quality of life [19].

Anemia of chronic kidney diseases is a state of anemia caused by various factors that interfere with the production and metabolism of RBC [20]. Patients with uremia have significant toxin accumulation, and most of the toxins can be effectively removed by PD [21]. However, some of the medium and large molecules may remain and inhibit the function of renal erythropoietin [22]. The inhibited synthesis of erythropoietin in the kidneys has a negative impact on the normal hematopoietic process [23], including reduced hemoglobin, RBC and TIBC [24].

The concentrations of hemoglobin, RBC and TIBC in the peripheral blood of the participants in both groups were compared. We found that, compared with the HC group, hemoglobin, RBC and TIBC concentrations in the peripheral blood were significantly lower in the PD group. Decreased anemia-related indexes in PD patients confirmed the existence of a certain degree of anemia. In patients with PD, those with high 25(OH)D concentration showed significantly higher concentrations of hemoglobin, RBC and TIBC in the peripheral blood than those with low 25(OH)D concentration. The Spearman correlation analysis further revealed that the concentrations of hemoglobin, RBC and TIBC in the peripheral blood of patients with PD were positively correlated with the concentrations of 25(OH)D, indicating that 25(OH)D concentration could reflect the severity of anemia.

The microinflammatory state refers to the stimulation of multiple inflammatory factors by toxin production and prolonged presence in the blood circulation, resulting in mild inflammation [25, 26]. The microinflammatory state is commonly found in various chronic kidney diseases and may increase the risk of cardiovascular events in the long term [27, 28]. Hs-CRP, IL-6 and TNF-α are molecules that are closely associated with the inflammatory response. CRP is synthesized by hepatocytes and is closely related to the process of inflammatory response, while IL-6 and TNF-α are involved in the regulation of inflammatory cell activation and infiltration during the inflammatory response [29].

We found that, compared with the HC group, serum concentrations of hs-CRP, IL-6 and TNF-α in the PD group were significantly higher, suggesting the presence of microinflammatory state in PD patients. In patients with PD, those with high 25(OH)D concentration showed significantly lower concentrations of hs-CRP, IL-6 and TNF-α in the peripheral blood than those with low 25(OH)D concentration. The Spearman correlation analysis further revealed that 25(OH)D concentration in PD patients was negatively correlated with hs-CRP, IL-6 and TNF-α concentrations. These results indicated that 25(OH)D showed significant negative correlation with microinflammatory status in the body.

The decreased antioxidants and increased toxins can lead to enhanced oxidative stress [30]. The presence of large amounts of toxins in the body of PD patients can directly stimulate the generation of reactive oxygen species and enhance lipid peroxidation, while decreased antioxidants may also exacerbate oxidative stress [31]. The indicator of oxidative stress, MDA, and the antioxidant indicators SOD and GSH-Px, were therefore analyzed in this research.

In this study, we compared the differences in the serum concentrations of oxygenation indicators MDA and antioxidant indicators SOD and GSH-Px between the two groups. We found that, compared with the HC group, MDA concentration was higher and SOD and GSH-Px concentrations were lower in the PD group. These results indicated that there was still an oxidative/antioxidative imbalance in the PD group. In the PD group, those with high 25(OH)D concentration showed lower MDA concentration and higher SOD and GSH-Px concentrations than those with low 25(OH)D concentration. The Spearman correlation analysis further revealed that 25(OH)D concentration in PD patients was negatively correlated with MDA concentration, and positively correlated with SOD and GSH-Px concentrations. Thus, 25(OH)D concentration could indicate oxidative stress in patients.

There were some limitations in this research that should be mentioned. First, serum 25(OH)D concentrations were not monitored over an extended duration. Future research should be designed to evaluate serum 25(OH)D concentrations and the other parameters over a longer study period in PD patients. Second, the mechanism of vitamin D action in PD patients was not investigated in this research. Third, the effects of the supplementation of vitamin D on anemia, oxidative stress and microinflammatory state in PD patients should be explored in future work. Although vitamin D has been used in the treatment of PD patients, vitamin D toxicity is a potential risk [32, 33]. Several studies have investigated the influence of vitamin D3 supplementation on serum 25(OH)D concentration and indicated the safe dose of vitamin D3 ranging from 5000 to 50,000 IUs/day [34–36]. Plasma 25(OH)D concentrations can be increased to 30–40 ng/mL by vitamin D3 supplementation, and changes in the corresponding symptoms in patients before and after vitamin D3 supplementation should be observed and analyzed.

## Conclusion

In conclusion, PD patients have shown low 25(OH)D concentration in the peripheral blood. The presence of reduced 25(OH)D concentrations in PD patients is related to anemia, oxidative stress and microinflammatory state. The supplementation of vitamin D may be a reliable way to optimize the overall status and to promote the desired therapeutic effect of patients with PD.

## Supplementary Information

**Additional file 1: Figure S1. **Inclusion and exclusion criteria for the selection of the patients. **Table S1.** Digital values for the figures.

## Data Availability

All data generated or analysed during this study are included in this published article.

## Abbreviations

PD: Peritoneal dialysis
HC: Healthy control
RBC: Blood cell count
TIBC: Total iron binding capacity

## References

[1] Mehrotra R, Devuyst O, Davies SJ, Johnson DW (2016). The current state of peritoneal dialysis. J Am Soc Nephrol.

[2] Borzych-Duzalka D, Aki TF, Azocar M, White C, Harvey E, Mir S, Adragna M, Serdaroglu E, Sinha R, Samaille C (2017). Peritoneal dialysis access revision in children: causes, interventions, and outcomes. Clin J Am Soc Nephrol.

[3] Planas JT (2012). Fluid transport and homeostasis in peritoneal dialysis. Contrib Nephrol.

[4] Stepanova N, Korol L, Burdeyna O (2019). Oxidative stress in peritoneal dialysis patients: association with the dialysis adequacy and technique survival. Indian J Nephrol.

[5] Zhu N, Yuan W, Zhou Y, Liu J, Bao J, Hao J, Miao W (2011). High mobility group box protein-1 correlates with microinflammatory state and nutritional status in continuous ambulatory peritoneal dialysis patients. J Artif Organs.

[6] Merino A, Portoles J, Selgas R, Ojeda R, Buendia P, Ocana J, Bajo MA, del Peso G, Carracedo J, Ramirez R (2010). Effect of different dialysis modalities on microinflammatory status and endothelial damage. Clin J Am Soc Nephrol.

[7] Collins AJ (2003). Cardiovascular mortality in end-stage renal disease. Am J Med Sci.

[8] Ahmadmehrabi S, Tang WHW (2018). Hemodialysis-induced cardiovascular disease. Semin Dial.

[9] Alwakeel JS, Usama S, Mitwalli AH, Alsuwaida A, Alghonaim M (2014). Prevalence of vitamin D deficiency in peritoneal dialysis patients. Saudi J Kidney Dis Transpl.

[10] My Thuc LT, Dung NQ, Ha VN, Tam ND, Hang Nga NT (2019). Actual diet and nutritional deficiencies status in children on peritoneal dialysis at the Vietnam National Hospital of Pediatrics. Saudi J Kidney Dis Transpl.

[11] Lopez RO, de Motta EE, Carmona A, Montemayor VG, Berdud I, Malo AM, Garcia PA (2018). Correction of 25-OH-vitamin D deficiency improves control of secondary hyperparathyroidism and reduces the inflammation in stable haemodialysis patients. Nefrologia.

[12] Pi HC, Ren YP, Wang Q, Xu R, Dong J (2015). Serum 25-hydroxyvitamin D level could predict the risk for peritoneal dialysis-associated peritonitis. Perit Dial Int.

[13] Singer RF (2013). Vitamin D in dialysis: defining deficiency and rationale for supplementation. Semin Dial.

[14] Sterling KA, Eftekhari P, Girndt M, Kimmel PL, Raj DS (2012). The immunoregulatory function of vitamin D: implications in chronic kidney disease. Nat Rev Nephrol.

[15] Hewison M (2011). Antibacterial effects of vitamin D. Nat Rev Endocrinol.

[16] Lehouck A, Mathieu C, Carremans C, Baeke F, Verhaegen J, Van Eldere J, Decallonne B, Bouillon R, Decramer M, Janssens W (2012). High doses of vitamin D to reduce exacerbations in chronic obstructive pulmonary disease: a randomized trial. Ann Intern Med.

[17] Saab G, Young DO, Gincherman Y, Giles K, Norwood K, Coyne DW (2007). Prevalence of vitamin D deficiency and the safety and effectiveness of monthly ergocalciferol in hemodialysis patients. Nephron Clin Pract.

[18] Kendrick J, Targher G, Smits G, Chonchol M (2009). 25-Hydroxyvitamin D deficiency and inflammation and their association with hemoglobin levels in chronic kidney disease. Am J Nephrol.

[19] Yuksel E, Aydin E (2021). The relationship between serum vitamin D levels and health-related quality of life in peritoneal dialysis patients. Int Urol Nephrol.

[20] Nangaku M, Eckardt KU (2006). Pathogenesis of renal anemia. Semin Nephrol.

[21] Ronco C, Dell'Aquila R, Rodighiero MP, Di Loreto P, Spano E (2006). Integration of peritoneal dialysis in the treatment of uremia. Contrib Nephrol.

[22] Sakaguchi Y, Hamano T, Wada A, Masakane I (2019). Types of erythropoietin-stimulating agents and mortality among patients undergoing hemodialysis. J Am Soc Nephrol.

[23] Palmer SC, Saglimbene V, Mavridis D, Salanti G, Craig JC, Tonelli M, Wiebe N, Strippoli GF (2014). Erythropoiesis-stimulating agents for anaemia in adults with chronic kidney disease: a network meta-analysis. Cochrane Database Syst Rev.

[24] Koury MJ (2005). Erythropoietin: the story of hypoxia and a finely regulated hematopoietic hormone. Exp Hematol.

[25] Kaysen GA (2001). The microinflammatory state in uremia: causes and potential consequences. J Am Soc Nephrol.

[26] Zhou WX, Zheng WB, Huang XM, Zhang Y, Nie XZ, Li HB, He D, Xie LQ (2009). Effects of oxymatrine on microinflammatory state in patients undergoing continuous hemodialysis: a randomized controlled trial. Zhong Xi Yi Jie He Xue Bao.

[27] Cao C, Wan X, Chen Y, Wu W (2009). Metabolic factors and microinflammatory state promote kidney injury in type 2 diabetes mellitus patients. Ren Fail.

[28] Carmona A, Guerrero F, Jimenez MJ, Ariza F, Aguera ML, Obrero T, Noci V, Munoz-Castaneda JR, Rodriguez M, Soriano S (2020). Inflammation, senescence and microRNAs in chronic kidney disease. Front Cell Dev Biol.

[29] Shi L, Song J, Zhang X, Li Y, Li H (2013). Correlation between the microinflammatory state and left ventricular structural and functional changes in maintenance haemodialysis patients. Exp Ther Med.

[30] Daenen K, Andries A, Mekahli D, Van Schepdael A, Jouret F, Bammens B (2019). Oxidative stress in chronic kidney disease. Pediatr Nephrol.

[31] Roumeliotis S, Eleftheriadis T, Liakopoulos V (2019). Is oxidative stress an issue in peritoneal dialysis?. Semin Dial.

[32] Weissheimer R, Bucharles SGE, Truyts CAM, Jorgetti V, Figueiredo AE, Barrett P, Olandoski M, Pecoits-Filho R, Moraes TP (2021). High prevalence of biochemical disturbances of chronic kidney disease—mineral and bone disorders (CKD-MBD) in a nation-wide peritoneal dialysis cohort: are guideline goals too hard to achieve?. J Bras Nefrol.

[33] Feghali K, Papamarkakis K, Clark J, Malhotra N, Stoddart L, Osakwe I (2021). Vitamin D toxicity managed with peritoneal dialysis. Case Rep Endocrinol.

[34] Bezrati I, Ben Fradj MK, Hammami R, Ouerghi N, Padulo J, Feki M (2020). A single mega dose of vitamin D3 improves selected physical variables in vitamin D-deficient young amateur soccer players: a randomized controlled trial. Appl Physiol Nutr Metab.

[35] Shirvani A, Kalajian TA, Song A, Allen R, Charoenngam N, Lewanczuk R, Holick MF (2020). Variable genomic and metabolomic responses to varying doses of vitamin D supplementation. Anticancer Res.

[36] McCullough PJ, Lehrer DS, Amend J (2019). Daily oral dosing of vitamin D3 using 5000 TO 50,000 international units a day in long-term hospitalized patients: insights from a seven year experience. J Steroid Biochem Mol Biol.

