# Supplementary material for: Low serum vitamin D concentration is correlated with anemia, microinflammation, and oxidative stress in patients with peritoneal dialysis
Source: J Transl Med. 2021 Sep 27;19:411. doi: 10.1186/s12967-021-03077-w (PMC8477502; doi:10.1186/s12967-021-03077-w)
Supplement: Supplementary file 1 — Additional file 1: Figure S1. Inclusion and exclusion criteria for the selection of the patients. Table S1. Digital values for the figures. [file 12967_2021_3077_MOESM1_ESM.docx]

**Additional file 1**


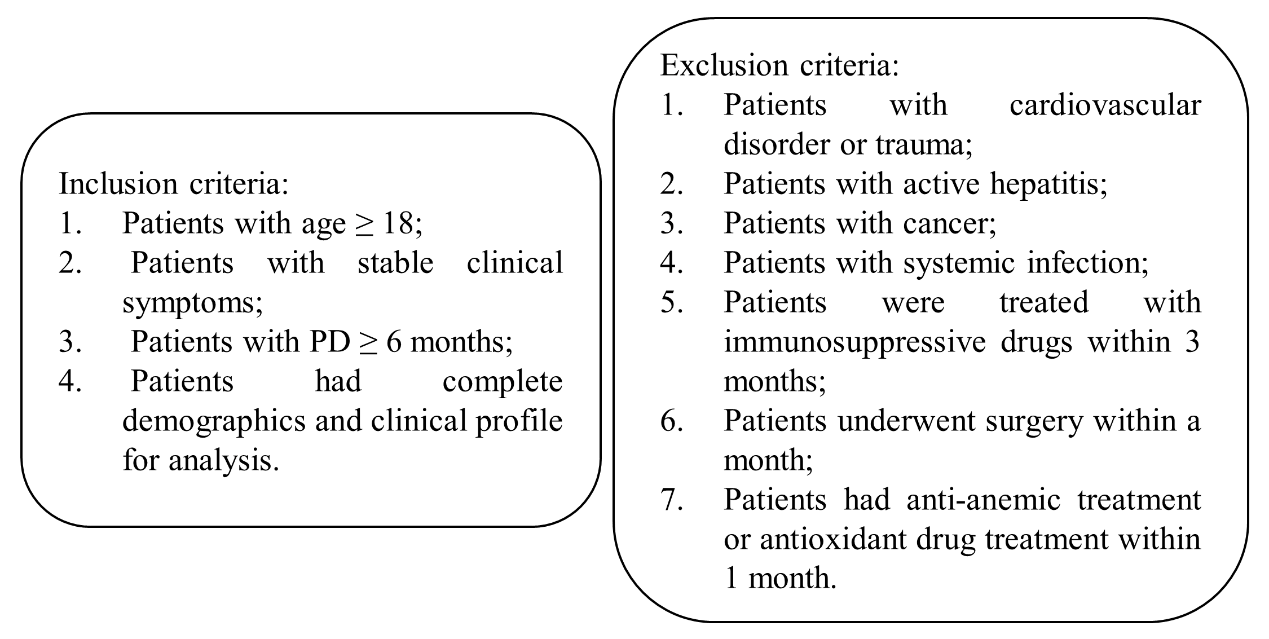


**Figure S1.** Inclusion and exclusion criteria for the selection of the patients.

**Table S1**. Digital values for the figures.

1. Digital values for Figure 2. (Median with interquartile range)

|  | HC (n = 56) | PD (n = 62) |
| --- | --- | --- |
| A | 133 (111 to 153) | 109 (96 to 128) |
| B | 4.5 (3.7 to 5.1) | 3.5 (3 to 4) |
| C | 58 (49 to 67) | 49 (39 to 55) |

1. Digital values for Figure 3. (Median with interquartile range)

|  | HC (n = 56) | PD (n = 62) |
| --- | --- | --- |
| A | 3.6 (2.7 to 4.5) | 7 (5.3 to 8.7) |
| B | 35 (24 to 45) | 88 (64 to 103) |
| C | 20 (12 to 36) | 56 (42 to 71) |

1. Digital values for Figure 4. (Median with interquartile range)

|  | HC (n = 56) | PD (n = 62) |
| --- | --- | --- |
| A | 4.2 (3.2 to 4.9) | 8.1 (6.5 to 10) |
| B | 102 (92 to 114) | 79 (66 to 99) |
| C | 93 (81 to 109) | 76 (66 to 97) |

1. Digital values for Figure 5. (Median with interquartile range)

|  | < 17.93 | > 17.93 |
| --- | --- | --- |
| A | 100 (92 to 117) | 120 (109 to 135) |
| B | 3.4 (2.9 to 3.8) | 3.7 (3.2 to 4.5) |
| C | 44 (37 to 51) | 54 (47 to 62) |
| D | 7.1 (6.2 to 9.1) | 6.1 (4.5 to 7.7) |
| E | 98 (77 to 106) | 76 (60 to 87) |
| F | 69 (52 to 78) | 44 (32 to 57) |
| G | 8.8 (7.2 to 10.5) | 7.6 (5.3 to 8.4) |
| H | 72 (64 to 87) | 88 (72 to 108) |
| I | 71 (58 to 87) | 94 (76 to 108) |
